# Systemic Antibiotics as Adjuncts in Periodontal Therapy for Stage III and Stage IV Periodontitis: An Umbrella Review Aligned with the 2018 World Workshop Classification, with AMSTAR-2 Critical Appraisal

**DOI:** 10.3390/antibiotics15090918

**Published:** 2026-09-17

**Authors:** Manchala Sesha Reddy, Mohammad Akheel, Mohammed Shoaib ur Rahman Tippu, Meharunneesa Aboobacker Sidheeq, Ban Al Mudarris, Syed Kuduruthullah, Sura Ali Ahmed Fuoad Al-Bayati, Brek Mohammed Brek Al Ameri, Nader Nabil Fouad Rezallah

**Affiliations:** 1 City University Ajman, Ajman P.O. Box 18484, United Arab Emirates; a.meharunneesa@cu.ac.ae (M.A.S.); b.mudarris@cu.ac.ae (B.A.M.); k.syed@cu.ac.ae (S.K.); brekalameri0@gmail.com (B.M.B.A.A.); n.nabil@cu.ac.ae (N.N.F.R.); 2 Oral & Maxillofacial Surgery, City University Ajman, Ajman P.O. Box 18484, United Arab Emirates; m.akheel@cu.ac.ae; 3 Gulf Medical University, Ajman P.O. Box 4181, United Arab Emirates; dr.shoaib@gmu.ac.ae (M.S.u.R.T.); dr.sura@gmu.ac.ae (S.A.A.F.A.-B.)

**Keywords:** Stage III periodontitis, Stage IV periodontitis, 2018 World Workshop classification, adjunctive systemic antibiotics, AMSTAR-2, non-surgical periodontal therapy, scaling and root planing, amoxicillin, metronidazole, azithromycin, doxycycline

## Abstract

**Introduction:** Severe periodontitis, corresponding to Stage III and Stage IV disease according to the 2018 World Workshop classification, represents a substantial global health burden. Systemic antibiotics have been investigated as adjuncts to mechanical periodontal therapy; however, their effectiveness and appropriate use in severe periodontitis remain uncertain. This umbrella review aimed to synthesize the available evidence on adjunctive systemic antibiotics in Stage III and Stage IV periodontitis and assess the methodological quality of the available systematic reviews. **Methods:** This umbrella review was conducted according to the Preferred Reporting Items for Systematic Reviews and Meta-Analyses (PRISMA) guidelines. A systematic search of MEDLINE, Embase, and other relevant databases was conducted from June 2018 to June 2026. Systematic reviews evaluating adjunctive systemic antibiotics in patients with Stage III or Stage IV periodontitis, classified according to the 2018 World Workshop, were eligible. Methodological quality was assessed using AMSTAR-2 and overlap among primary studies was evaluated using the Corrected Covered Area (CCA). **Results:** Two systematic reviews met the inclusion criteria: a network meta-analysis by Lin et al. and a systematic review by Jones and Hoyle focusing on azithromycin. The CCA was approximately 52.9%, indicating substantial overlap between the included reviews. Lin et al. reported moderate-certainty evidence that adjunctive amoxicillin plus metronidazole (AMX + MTZ) resulted in greater clinical attachment level gain and probing pocket depth reduction compared with mechanical debridement alone. Jones and Hoyle found insufficient evidence to support routine adjunctive azithromycin, owing to low certainty and heterogeneity among studies. **Discussion and Conclusions**: Adjunctive systemic antibiotics, particularly AMX + MTZ, may provide additional clinical benefits in severe periodontitis. However, the limited number of systematic reviews, substantial overlaps, and uncertainty surrounding some antibiotic protocols limit the strength of the evidence. Current findings support selective rather than routine antibiotic use, guided by individual patient and disease characteristics and antimicrobial stewardship. Future studies should emphasize long-term outcomes and consistently distinguish Stage III from Stage IV periodontitis.

## 1. Introduction

Periodontitis is a chronic inflammatory disease that leads to the progressive destruction of tooth-supporting tissues. It is characterized by clinical attachment loss, alveolar bone resorption, periodontal pocket formation, and bleeding on probing. If not diagnosed and managed appropriately, the disease can eventually result in tooth loss, despite being largely preventable and treatable through timely intervention and appropriate periodontal care [1]. Under the 2018 World Workshop classification, severe forms of the disease are classified as Stage III and Stage IV periodontitis, both of which are associated with substantial functional impairment and contribute significantly to the global burden of oral disease [2].

Severe periodontitis, classified as Stage III and Stage IV under the 2018 World Workshop classification, represents a major global public health challenge. In 2021, approximately 1.07 billion people worldwide were affected, corresponding to an age-standardized prevalence of 12.5%, while 353 million individuals were edentulous. The burden continues to increase because of population growth and ageing and is projected to exceed 1.5 billion cases by 2050 [2,3]. Population-based studies also demonstrate a high prevalence of Stage III and Stage IV periodontitis disease in different regions, including Turkey, China, and West Africa, highlighting its widespread clinical and public health impact [4,5,6].

The 2018 World Workshop classification introduced a staging and grading framework that characterizes disease severity, complexity, and risk of progression, thereby providing a more comprehensive basis for diagnosis, treatment planning, and prognosis than the previous chronic and aggressive periodontitis classifications. Stage III and Stage IV periodontitis are defined by interdental clinical attachment loss (CAL) of ≥5 mm and radiographic bone loss extending to the middle third of the root or beyond, while Stage IV is further distinguished by complexity factors such as masticatory dysfunction, pathologic tooth migration, bite collapse, and extensive tooth loss requiring multidisciplinary rehabilitation. Disease grade (A, B, or C) reflects the estimated rate of progression, with Grade C representing rapidly progressing disease. This contemporary classification provides the framework for current evidence-based clinical practice and forms the basis for the present umbrella review [7,8].

The European Federation of Periodontology (EFP) S3 clinical practice guidelines provide an evidence-based framework for the management of periodontitis, integrating the best available evidence with expert consensus [9,10]. Although their implementation may be limited in some healthcare settings by workforce and economic constraints [11,12,13], the guidelines consistently advise against the routine use of adjunctive systemic antibiotics during non-surgical periodontal therapy. Instead, antibiotic therapy should be reserved for carefully selected patients after considering disease severity, the expected clinical benefit, and the principles of antimicrobial stewardship. For Stage IV periodontitis, the periodontal component of treatment follows the same evidence-based recommendations as Stage I–III disease [9,10]. Non-surgical periodontal therapy, comprising supra- and subgingival instrumentation performed either quadrant-wise or as full-mouth treatment, remains the cornerstone of periodontitis management [1,7].

Patients with stage III and IV periodontitis exhibit advanced periodontal destruction and are the most likely candidates for adjunctive systemic antimicrobial therapy, particularly when mechanical debridement is completed within a short time frame to minimize bacterial recolonization [10,14]. The management of Stage IV periodontitis requires a multidisciplinary, individualised approach that integrates periodontal therapy with functional rehabilitation and lifelong supportive periodontal care. The periodontal treatment component should follow the evidence-based EFP S3 guidelines for Stage I–III periodontitis [9].

The use of systemic antibiotics as adjuncts to non-surgical periodontal therapy (NSPT) has been investigated since the late 1980s. Early clinical studies from the Leuven group demonstrated that full-mouth scaling and root planing combined with amoxicillin and metronidazole (AMX + MTZ) produced greater improvements in probing depth reduction and clinical attachment gain than mechanical debridement alone, particularly in advanced periodontitis [14,15,16].

Recent observational evidence has continued to evaluate the real-world effectiveness of adjunctive systemic antibiotics, with cohort data suggesting variable clinical responses following non-surgical periodontal therapy [17]. More recently, an umbrella review synthesizing 44 systematic reviews and 221 meta-analyses concluded that the certainty of evidence supporting routine adjunctive antibiotic use remains limited because of the generally low methodological quality of the available reviews, despite many pooled estimates being relatively stable [18].

Current evidence suggests that a uniform approach to adjunctive systemic antibiotic use is inappropriate, and that antibiotic selection should be guided by the best available evidence and individual patient characteristics. Interpreting the available evidence is challenging because of the considerable heterogeneity across studies. Differences in patient characteristics, disease severity, antibiotic regimens, follow-up duration, and outcome measures make direct comparisons difficult and may mask clinically important benefits in specific patient groups, particularly those with severe periodontitis [19]. Another key challenge is identifying the patients who are most likely to benefit from adjunctive systemic antibiotics, as current evidence does not support their routine use in all cases [20,21].

Several systematic reviews have evaluated systemic antibiotics as adjuncts to periodontal therapy, but their findings vary because of differences in antibiotic regimens, patient populations, disease classifications, and analytical methods. An umbrella review can bring this evidence together, assess the quality and overlap of existing reviews, and determine how consistent and reliable their conclusions are. This is particularly relevant under the 2018 World Workshop classification, which provides a contemporary framework for assessing disease severity and complexity.

Although adjunctive systemic antibiotics may improve periodontal treatment outcomes, substantial heterogeneity in antibiotic regimens, treatment protocols, and study designs limits the consistency of evidence. Consequently, high-quality evidence synthesis is essential to provide a comprehensive and reliable overview of their clinical effectiveness. This umbrella review addresses this gap by restricting inclusion to systematic reviews and meta-analyses based on the 2018 World Workshop classification and assessing their methodological quality using AMSTAR-2 [22] while evaluating overlap among the included reviews. The review aims to synthesize the available evidence on adjunctive systemic antibiotics in Stage III and Stage IV periodontitis, inform clinical decision-making, and identify priorities for future research.

## 2. Materials and Methods

The review was prospectively registered in PROSPERO (CRD420261449383) and reported in accordance with the PRISMA guidelines [23].

### 2.1. Eligibility Criteria

Only articles published in English were included; studies published in other languages were excluded. Published systematic reviews and meta-analyses were eligible, whereas conference abstracts, protocols, editorials, letters, and other non-peer-reviewed publications were excluded. Systematic reviews without a quantitative meta-analysis were excluded according to the predefined eligibility criteria.

Where multiple systematic reviews included overlapping primary studies, all eligible reviews were initially retained, and the extent of overlap was subsequently quantified using the Corrected Covered Area (CCA). The presence of overlapping primary studies was considered during interpretation to avoid treating evidence derived from the same trials as independent evidence. The CCA was calculated using the standard formula: CCA = (N − r)/(rc − r), where N represents the total number of study occurrences across the included reviews, r is the number of reviews, and c is the number of unique primary studies.

### 2.2. The Eligibility Criteria Were Structured According to the PICO Framework

Population (P): Adults diagnosed with Stage III or Stage IV periodontitis per the 2018 World Workshop classification.

Intervention (I): Systemic antibiotics administered adjunctive to non-surgical periodontal therapy, specifically focusing on amoxicillin plus metronidazole (AMX + MTZ), azithromycin, and doxycycline.

Comparator (C): Non-surgical periodontal therapy alone, including scaling and root planing or full-mouth debridement, performed with or without the administration of a placebo.

Outcomes (O): Clinical attachment level, probing pocket depth, bleeding on probing, microbiological outcomes, and the incidence of adverse events.

The population comprised adults diagnosed with Stage III or Stage IV periodontitis according to the 2018 World Workshop classification. The intervention was systemic antibiotic therapy administered as an adjunct to non-surgical periodontal therapy, with particular consideration of amoxicillin plus metronidazole (AMX + MTZ), azithromycin, and doxycycline. The comparator was non-surgical periodontal therapy alone, including scaling and root planing or full-mouth debridement, with or without placebo. The principal outcomes were clinical attachment level, probing pocket depth, bleeding on probing, microbiological outcomes, and adverse events.

### 2.3. Information Sources and Search Strategy

The electronic databases searched were Embase, Google Scholar, LILACS, MEDLINE, PubMed, and Scopus. Searches were conducted from 1 June 2018 to 1 June 2026. Grey literature was additionally searched through OpenGrey, and reference lists of the included reviews were manually screened for potentially eligible publications. The search strategy included terms relating to Stage III and Stage IV periodontitis, the 2018 World Workshop classification, adjunctive systemic antibiotics, non-surgical periodontal therapy, scaling and root planing, amoxicillin, metronidazole, azithromycin, doxycycline, and AMSTAR-2. AMSTAR-2 Assessment: Two reviewers independently assessed the methodological quality of each included review using all 16 AMSTAR-2 domains, with particular consideration of the seven critical domains. Reviewer judgments were compared, and disagreements were resolved by consensus or consultation with a third reviewer when required. The methodological quality of the included systematic reviews was assessed using AMSTAR 2, with overall confidence ratings based on weaknesses in critical domains, as summarized in Table 1.

### 2.4. Study Selection and Data Extraction

Two independent reviewers (S.R. and M.A.) screened the identified systematic reviews and meta-analyses. Reviews evaluating human studies of adjunctive systemic antibiotic therapy were considered eligible. Narrative reviews, umbrella reviews, studies without appropriate pooled estimates or comparators, and duplicate secondary analyses were excluded. Data were independently extracted using a standardized form covering study characteristics, participant characteristics, antibiotic regimens, outcomes, methodological quality, and reported effect estimates. Disagreements were resolved through discussion and, when necessary, consultation with a third reviewer.

### 2.5. Methodological Quality and Certainty of Evidence

The methodological quality of the included systematic reviews was independently assessed by two reviewers using AMSTAR-2. The assessment considered all applicable AMSTAR-2 domains, including the critical domains specified by the instrument. Disagreements between reviewers were resolved by consensus, with consultation with a third reviewer when required. The overall confidence in each review was interpreted in accordance with AMSTAR-2 guidance, with particular attention to the critical domains.

The certainty of the meta-analytic evidence reported by the included reviews was extracted where available. Certainty was interpreted according to the GRADE assessments reported by the original reviews.

### 2.6. Overlap Assessment

Because umbrella reviews may contain systematic reviews based on overlapping primary studies, overlap was assessed using the Corrected Covered Area (CCA) method [26]. The CCA was calculated from the citation matrix of primary studies included across the eligible systematic reviews. The degree of overlap was considered when interpreting the overall evidence to avoid giving disproportionate weight to evidence derived from the same primary trials.

### 2.7. Data Synthesis

The findings of the included systematic reviews were synthesized narratively according to antibiotic regimen, clinical outcomes, methodological quality, certainty of evidence, and overlap between reviews. Because only two reviews met the eligibility criteria and their intervention and analytical scopes differed, no additional quantitative pooling was performed at the umbrella-review level.

## 3. Results

### 3.1. Study Selection

**Figure 1:**
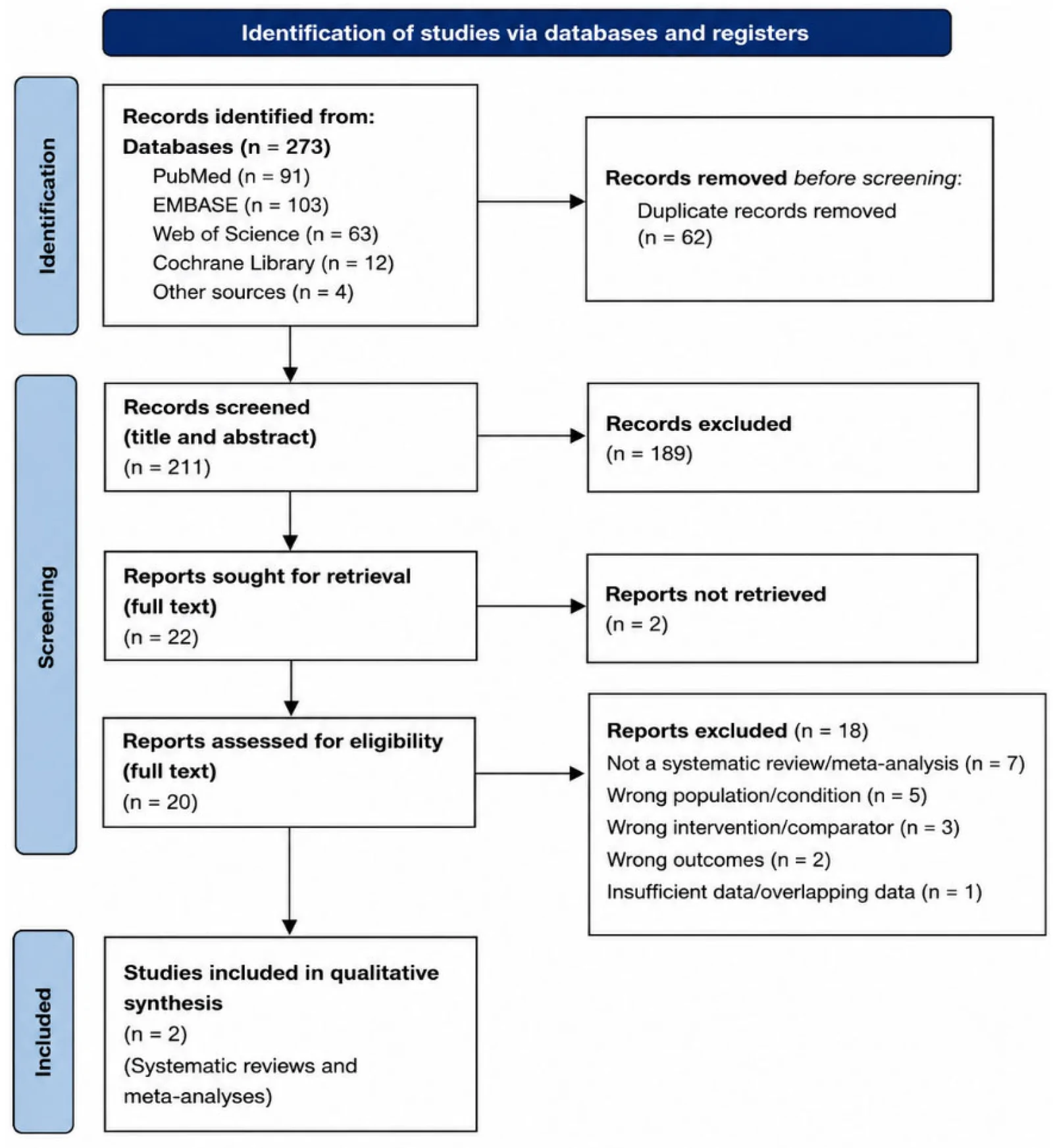
PRISMA 2020 flow diagram of study selection.

The literature search yielded 273 records. After removal of duplicate records, the remaining citations underwent title and abstract screening, followed by full-text assessment against the predefined eligibility criteria. Two reviewers independently performed the selection process, with disagreements resolved through discussion and consensus. Two systematic reviews and meta-analyses met the eligibility criteria and were included in the qualitative synthesis. The study-selection process is presented in the PRISMA 2020 flow diagram (Figure 1).

### 3.2. Characteristics of the Included Reviews

The two included systematic reviews examined adjunctive systemic antibiotics in patients with advanced periodontitis classified according to the 2018 World Workshop framework. Published between 2022 and 2025, the reviews evaluated antibiotic regimens administered in conjunction with non-surgical periodontal therapy, including amoxicillin plus metronidazole (AMX + MTZ), azithromycin, and doxycycline.

**Methodological Quality Assessment:** Lin et al. (2025) [24] evaluated multiple systemic antibiotic regimens and included approximately 45 randomized controlled trials. The review used comparative approaches, including network meta-analysis, to assess the relative effectiveness of different antibiotic strategies. The principal outcomes included clinical attachment level (CAL), probing pocket depth (PPD), bleeding on probing (BOP), microbiological outcomes, and adverse events.

Jones and Hoyle (2022) [25] focused primarily on azithromycin and included a smaller number of randomized controlled trials. The authors used conventional pairwise meta-analysis and primarily assessed periodontal clinical outcomes following adjunctive azithromycin therapy.

Although the two reviews differed in scope, intervention focus, and analytical approach, both evaluated whether systemic antibiotics provided additional benefits beyond mechanical periodontal therapy. Table 2. Narrative Comparison of the Characteristics and Main Findings of the Included Systematic Reviews. A detailed comparison of their characteristics and key findings is presented in Table 2.

### 3.3. Methodological Quality Assessment

Both included reviews reported several methodological strengths, including registered protocols, comprehensive literature searches, duplicate study selection, risk-of-bias assessment, assessment of publication bias, and evaluation of certainty of evidence. Lin et al. [24] provided a broader assessment because multiple antibiotic regimens were considered using comparative analytical methods, whereas Jones and Hoyle [25] focused specifically on azithromycin. The methodological quality assessments are summarized in Table 1.

### 3.4. Overlap Between Included Reviews

Considerable overlap was identified between the two systematic reviews. For the two included reviews, N was 56, r = 2, and c was 52. The CCA was calculated as (56 − 2)/[(2 × 54) − 2] × 100, yielding a CCA of 52.9%, indicating a substantial overlap in the underlying primary studies. The overlapping primary studies and detailed CCA calculation are presented in Table 3. Several randomized controlled trials contributed evidence to both reviews. This overlap was considered when interpreting the findings because treating the same primary trials as independent evidence could exaggerate the apparent strength or consistency of the evidence.

### 3.5. Comparative Effectiveness of Antibiotic Regimens

**Amoxicillin Plus Metronidazole:** AMX + MTZ showed the most consistent evidence of adjunctive clinical benefit among the antibiotic regimens evaluated (Table 4). Comparison of Adjunctive Systemic Antibiotic Regimens Evaluated in the Included Systematic Reviews. In the review by Lin et al. [24], the combination was associated with greater improvements in CAL and PPD when administered alongside non-surgical periodontal therapy (Table 4).

**Azithromycin:** The evidence for azithromycin was less consistent. Some studies demonstrated short-term clinical improvements, but interpretation was limited by the small number of trials, differences in treatment protocols, and uncertainty regarding superiority over non-surgical periodontal therapy alone. Jones and Hoyle [25] did not support routine adjunctive use of azithromycin.

**Doxycycline:** Evidence concerning adjunctive doxycycline was limited, preventing meaningful comparison with the other antibiotic regimens. Its effectiveness in patients classified as having Stage III or Stage IV periodontitis therefore remains uncertain.

**Certainty of Evidence:** The certainty of evidence reported by the included systematic reviews varied according to outcome and review methodology. Lin et al. [24] reported moderate certainty for key outcomes including CAL and PPD, with downgrading related to variability in antibiotic regimens, follow-up periods, and patient characteristics. Jones and Hoyle [25] assigned lower certainty to several outcomes because of small sample sizes, methodological limitations, heterogeneity, and inconsistent treatment effects. Table 5. Certainty of Evidence (GRADE) for Clinical Outcomes Reported in the Included Systematic Reviews. The greatest uncertainty was observed for BOP, which was rated as very low certainty in the Jones and Hoyle review (Table 5).

## 4. Discussion

This umbrella review identified only two systematic reviews that specifically address adjunctive systemic antibiotics in periodontitis, classified according to the 2018 World Workshop framework. The limited number of eligible reviews is important to consider when interpreting the findings, because the available evidence base remains relatively narrow despite the larger body of literature on systemic antibiotics published under earlier disease classifications.

The 52.9% CCA indicates considerable overlap among the included reviews, with several primary studies appearing more than once. Therefore, agreement between reviews should not be viewed as independent confirmation of the evidence. Repeated inclusion of the same trials may give undue weight to certain findings.

Interpretation should therefore focus on the quality and characteristics of the underlying studies.

The principal finding is that the evidence does not support a uniform approach to adjunctive antibiotic therapy. Differences in antibiotic regimens, patient characteristics, treatment protocols, and methodological quality contribute to variation in the observed effects. The apparent advantage of AMX + MTZ should therefore be interpreted as evidence of greater support relative to the other regimens evaluated, rather than as evidence that the combination should be routinely prescribed [14,27,28,29].

The findings of this umbrella review are consistent with the EFP S3-level Clinical Practice Guideline, which advocates a selective rather than routine use of systemic antibiotics in the management of periodontitis. Table 6: Comparison of Umbrella Review Findings with EFP S3-Level Clinical Practice Guideline Recommendations. Both the guideline and the present review support the use of systemic antibiotics only in carefully selected patients who are likely to benefit from adjunctive therapy, emphasizing that antibiotics should complement, not replace, effective mechanical periodontal treatment and ongoing supportive periodontal care [9,10] (Table 6).

The substantial overlap between the reviews included also affects interpretation. A CCA of approximately 52.9% indicates that a considerable proportion of the underlying primary evidence was shared between the reviews. Consequently, the apparent consistency of findings should not be interpreted as representing independent confirmation from two entirely separate evidence bases. The conclusions of the umbrella review were therefore interpreted at the level of the underlying primary evidence rather than by simply counting the number of reviews supporting a particular intervention [28,30,31].

The evidence for azithromycin was less convincing, particularly given the limited number of trials and the methodological and clinical heterogeneity identified across studies. Similarly, the sparse evidence for doxycycline prevents firm conclusions regarding its role in patients classified under the 2018 staging system.

### 4.1. Stage III Versus Stage IV Evidence

An important consideration is the extent to which the available evidence represents true Stage IV periodontitis. Although the eligibility criteria included both Stage III and Stage IV disease, the underlying evidence appears to be dominated by patients with Stage III disease. Consequently, the findings should not be interpreted as demonstrating equivalent efficacy of adjunctive antibiotics in Stage IV periodontitis. This distinction is clinically relevant because Stage IV periodontitis is defined not only by severe periodontal destruction but also by additional complexity, including functional impairment, pathologic tooth migration, bite collapse, and extensive tooth loss. These characteristics may influence treatment planning, prognosis, and the feasibility of mechanical periodontal therapy. The paucity of Stage IV-specific evidence therefore represents a major limitation of the current evidence base and highlights the need for dedicated trials involving well-characterized Stage IV populations. The evidence for true Stage IV periodontitis remains limited, as few studies clearly included patients meeting the current Stage IV criteria. Therefore, these findings should be interpreted cautiously and may not be fully applicable to patients with more advanced and complex disease.

Taken together, these findings support a selective rather than routine approach to systemic antibiotic therapy. The clinical value of adjunctive antibiotics is likely to depend on appropriate patient selection and effective mechanical periodontal treatment rather than antibiotic prescription alone.

### 4.2. Antimicrobial Stewardship Considerations

Antimicrobial stewardship is central to decision-making regarding adjunctive systemic antibiotic therapy for periodontitis. The World Health Organization (WHO) framework emphasizes the need to optimize antimicrobial use while minimizing adverse effects and the emergence of antimicrobial resistance [21]. In periodontology, this requires balancing the potential clinical benefits of adjunctive systemic antibiotics against the risks associated with antibiotic exposure, including antimicrobial resistance, disruption of the oral microbiome, allergic reactions, gastrointestinal adverse effects, and unnecessary prescriptions [21,27,29]. The oral cavity is an important microbial reservoir, and antibiotic exposure may promote the selection and dissemination of resistant microorganisms [10,29]. Therefore, systemic antibiotics should be reserved for patients who are most likely to benefit, particularly those with advanced disease characteristics or an inadequate response to conventional non-surgical periodontal therapy [10,32].

This stewardship-based approach is further supported by a recent triple-blind randomized controlled trial by Ramachandra et al., which demonstrated that adjunctive azithromycin did not provide significant additional long-term clinical benefits compared with non-surgical periodontal therapy alone in patients with Stage III and Stage IV periodontitis, despite transient microbiological changes [33]. The current evidence therefore supports a stewardship-based approach to periodontal therapy that prioritizes optimal subgingival instrumentation and supportive periodontal maintenance, careful patient selection, the selective use of evidence-supported regimens such as amoxicillin plus metronidazole (AMX + MTZ) when clinically indicated, and avoidance of routine antibiotic prescribing without clear clinical justification [10,24,28,32].

### 4.3. Research Gaps and Future Directions

Despite the increasing evidence supporting adjunctive systemic antibiotics in periodontitis, several important knowledge gaps remain. A major limitation is the limited number of studies conducted using the 2018 World Workshop classification. Another important limitation is the predominance of short-term follow-up, with most studies reporting outcomes within 12 months. Consequently, evidence regarding the long-term effects of adjunctive systemic antibiotics on tooth retention, disease stability, recurrence, and supportive periodontal maintenance remains limited. Future research should also incorporate patient-centered outcomes, including oral health-related quality of life, functional outcomes, and patient satisfaction, in addition to conventional clinical measures such as clinical attachment level and probing pocket depth. Furthermore, few studies have evaluated the impact of antibiotic therapy on antimicrobial resistance, changes in the oral microbiome, or other long-term ecological consequences of antibiotic exposure.

Finally, substantial heterogeneity in antibiotic selection, dosage, treatment duration, and patient selection criteria continues to hinder direct comparisons across studies and the identification of optimal treatment protocols. Future research should therefore prioritize well-designed, adequately powered multicenter randomized controlled trials using standardized antibiotic regimens within the 2018 classification framework. In parallel, living systematic reviews should be considered to ensure that clinical recommendations remain current as new evidence continues to emerge. Limitations include the small number of eligible reviews, residual heterogeneity among primary studies, variability in antibiotic protocols, short follow-up periods, and limited reporting of adverse effects and antimicrobial resistance.

### 4.4. Strengths and Limitations

A key strength of this umbrella review is its prospective registration in PROSPERO and its focus on Stage III and Stage IV periodontitis defined according to the 2018 World Workshop classification. Restricting eligibility to the contemporary classification framework improves the clinical relevance of the findings and reduces heterogeneity associated with the use of outdated disease definitions.

However, several limitations should be considered. Only two systematic reviews met the eligibility criteria, substantially limiting the breadth of evidence available for synthesis. The underlying reviews also contained heterogeneous populations, antibiotic regimens, treatment protocols, and follow-up periods. The considerable overlap between the reviews included, reflected by a CCA of approximately 21%, further limits the independence of the available evidence and creates a risk that some primary trials may contribute disproportionately to the overall conclusions.

A further limitation is the limited representation of true Stage IV populations. Although the present review was designed to include both Stage III and Stage IV periodontitis, much of the available evidence appears to be derived from patients with Stage III disease. Therefore, the findings should not be interpreted as providing strong Stage IV-specific evidence. This distinction is important because Stage IV periodontitis is characterized by additional functional and restorative complexity.

Other limitations include residual heterogeneity among the primary studies, variation in antibiotic dosage and treatment duration, relatively short follow-up periods, and limited reporting of adverse effects and antimicrobial resistance. These factors restrict conclusions regarding long-term clinical benefits, tooth retention, recurrence, and ecological consequences of antibiotic exposure.

### 4.5. Clinical Implications

The findings do not support routine prescription of systemic antibiotics for patients with Stage III or Stage IV periodontitis. Although AMX + MTZ demonstrated the most consistent evidence of additional clinical benefit among the regimens evaluated, the magnitude of benefit should be interpreted cautiously given the limited number of eligible systematic reviews, overlap between reviews, heterogeneity among the underlying trials, and limited Stage IV-specific evidence.

Systemic antibiotics should be considered only for carefully selected patients in whom the anticipated clinical benefit is likely to outweigh the potential risks of antibiotic exposure. Optimal mechanical periodontal therapy remains the foundation of treatment and should be completed before considering adjunctive antibiotic therapy. Patient selection should take into account disease severity and complexity, progression risk, response to mechanical treatment, relevant patient factors, contraindications, and antimicrobial stewardship principles.

Accordingly, AMX + MTZ may be considered as an adjunct in appropriately selected patients when clinically justified, rather than as a routine component of non-surgical periodontal therapy. This interpretation is consistent with the EFP S3 clinical practice recommendations, which emphasize selective antibiotic use and the importance of effective mechanical periodontal treatment and supportive periodontal care.

## 5. Conclusions

This umbrella review found limited evidence that adjunctive systemic antibiotics provide additional clinical benefit in Stage III and Stage IV periodontitis. Among the evaluated regimens, amoxicillin plus metronidazole showed the most consistent evidence of benefit, although the clinical effect was modest and does not support routine antibiotic use. The confidence in these findings is reduced by overlap between the included systematic reviews, heterogeneity of the underlying studies, limited long-term evidence, and the small representation of patients with true Stage IV periodontitis. Mechanical periodontal therapy should therefore remain the basis of treatment, with systemic antibiotics considered only for selected patients and with antimicrobial stewardship in mind. Further well-designed studies classified according to the 2018 World Workshop criteria, particularly those reporting Stage IV-specific and long-term patient-centred outcomes, are needed to strengthen the evidence base.

## Figures and Tables

**Table 1:** Methodological Quality Assessment of Included Systematic Reviews Using the AMSTAR 2 Tool.

Items	Lin et al. (2025) [24]	Jones & Hoyle (2022) [25]
Item 1: PICO included	Yes	Yes
Item 2: Protocol registered	Yes	Yes
Item 3: Selection of the study designs	Yes	Yes
Item 4: Comprehensive search	Yes	Yes
Item 5: Study selection in duplicate	Yes	Yes
Item 6: Data extraction in duplicate	Yes	Yes
Item 7: List of excluded studies and justification of the exclusions	Yes	Yes
Item 8: Description of included studies	Yes	Yes
Item 9: Risk of bias assessed	Yes	Yes
Item 10: Reporting sources of funding for included studies	Yes	No information
Item 11: Appropriate meta-analytic methods	Yes	Yes
Item 12: Assessment of impact of risk of bias on meta-analysis	Yes	Yes
Item 13: Risk of bias considered when interpreting results	Yes	Yes
Item 14: Explanation of heterogeneity	Yes	Yes
Item 15: Publication bias assessment	Yes	Partly Yes
Item 16: Conflict of interest and funding of review	Yes	Yes
Overall rating	High	Moderate

**Table 2:** Narrative Comparison of the Characteristics and Main Findings of the Included Systematic Reviews.

Feature	Lin et al. 2025 [24]	Jones and Hoyle 2022 [25]
Population	Stage III/IV Grade C	Grade C
Intervention	Multiple adjuncts	Azithromycin only
Number of RCTs	50	6
Network MA	Yes	Conventional MA
Primary outcomes assessed	CAL, PPD, BOP, and other periodontal clinical parameters	Primarily clinical periodontal parameters following adjunctive azithromycin therapy
Main conclusion	AMX + MTZ best	AZM not superior

**Table 3:** Primary Study Overlap Between the Included Systematic Reviews and Corrected Covered Area (CCA) Analysis.

Primary Study	Lin et al. (2025) [24]	Jones & Hoyle (2022) [25]
Emingil et al. 2012	Yes	Yes
Haas et al. 2008	Yes	Yes
Ercan et al. 2015	No	Yes
Fujii et al. 2004	No	Yes
Haas 2012	Yes	Yes
Martande 2016	Yes	Yes
Total studies	50	6

**Table 4:** Comparison of Adjunctive Systemic Antibiotic Regimens Evaluated in the Included Systematic Reviews.

Antibiotic	Review	Recommendation	Timepoint	PD Reduction (WMD, mm) [95% CI]	CAL Gain (WMD, mm) [95% CI]
AMX + MTZ	Lin et al. (2025) [24]	Most effective	Short-term	0.74 (0.34–1.14)	0.82 (0.35–1.30)
			Medium-term	0.76 (0.21–1.30)	0.69 (0.01–1.37)
			Long-term	1.90 (1.32–2.48)	2.27 (0.40–4.14)
AMX + MTZ	Jones & Hoyle (2022) [25]		3 months	−0.39 (approximate)	Not reported
			6 months	−0.51 (approximate)	Not reported
			12 months	−0.51 (approximate)	Not reported
Azithromycin	Jones & Hoyle (2022) [25]	Similar short-term effect but insufficient evidence	3 months	−0.39 (−0.66, −0.13)	−0.61 (−1.13, −0.10)
			6 months	−0.88 (−2.10, 0.34)	−0.58 (−1.26, 0.10)
			12 months	−1.32 (−1.71, −0.93)	−0.88 (−1.32, −0.44)
Azithromycin	Lin et al. (2025) [24]		Short-term	0.56 (0.19–1.31)	0.56 (0.19–1.31)
			Medium-term	0.71 (0.06–1.35)	0.79 (0.02–1.56)
			Long-term	1.60 (0.87–2.33)	1.97 (0.09–3.84)
Doxycycline	Not included	Insufficient evidence			

**Table 5:** Certainty of Evidence (GRADE) for Clinical Outcomes Reported in the Included Systematic Reviews.

Outcome	Lin et al. (2025) [24]	Jones & Hoyle (2022) [25]	Explanation for Difference
CAL	Moderate	Low	Lin et al. assigned moderate certainty, indicating relatively consistent effects supported by reasonably conducted RCTs, with downgrading mainly due to variability in antibiotic regimens, follow-up periods, and patient characteristics.
			Jones et al. used a more conservative approach, downgrading certainty because of greater concerns regarding risk of bias, small sample sizes, imprecision, and heterogeneity among included studies.
PPD	Moderate	Low	Lin et al. maintained moderate certainty for PPD, considering the reduction consistent and clinically meaningful despite some variability.
			Jones et al. downgraded the evidence due to heterogeneity in treatment effects, differences in baseline disease severity, and uncertainty regarding the clinical importance of pooled PPD changes.
BOP	Moderate	Very Low	BOP showed the largest difference in certainty ratings. Lin et al. considered the evidence moderate certainty due to relatively consistent improvements in inflammatory outcomes, whereas
			Jones et al. rated it very low certainty because of concerns regarding risk of bias, heterogeneity, indirect outcome measurement, small sample sizes, and inconsistent reporting.

**Table 6:** Comparison of Umbrella Review Findings with the EFP S3-Level Clinical Practice Guideline Recommendations.

Guideline Recommendation	Evidence from Umbrella Review	Evidence from Umbrella Review
No routine antibiotics	Supported	Supported. The overall evidence indicates that adjunctive systemic antibiotics provide additional clinical benefits only in selected patients and do not justify routine prescription for all Stage III/IV periodontitis cases.
AMX + MTZ only for selected Stage III patients	Supported	Supported. Among evaluated antibiotic regimens, AMX + MTZ demonstrated the most consistent improvements in CAL and PPD, particularly in severe periodontal cases and patients with specific clinical characteristics suggesting higher potential benefit.
Antibiotic stewardship	Supported	Supported. The limited and variable magnitude of benefit, combined with concerns regarding resistance and adverse effects, reinforces the need for individualized prescribing decisions and avoidance of unnecessary antibiotic exposure.
Routine AZM	Not supported	Not supported. Evidence for azithromycin remains less consistent, with greater uncertainty regarding clinical benefit compared with AMX + MTZ. Current evidence does not support its routine use as an adjunctive therapy.

## Data Availability

No new datasets were generated or analyzed during this study. All data included in this umbrella review were obtained from previously published systematic reviews and are available from the corresponding publications.

## References

[1] Papapanou P.N., Sanz M., Buduneli N., Dietrich T., Feres M., Fine D.H., Flemmig T.F., Garcia R., Giannobile W.V., Graziani F. (2018). Periodontitis: Consensus Report of Workgroup 2 of the 2017 World Workshop on the Classification of Periodontal and Peri-Implant Diseases and Conditions. J. Periodontol..

[2] Nascimento G.G., Alves-Costa S., Romandini M. (2024). Burden of severe periodontitis and edentulism in 2021, with projections up to 2050: The Global Burden of Disease 2021 study. J. Periodontal Res..

[3] Wu L., Huang C.M., Wang Q., Wei J., Xie L., Hu C.Y. (2025). Burden of severe periodontitis: New insights based on a systematic analysis of the Global Burden of Disease Study 2021. BMC Oral Health.

[4] Germen M., Baser U., Lacin C.C., Fıratlı E., Issever H., Yalcin F. (2021). Periodontitis prevalence, severity, and risk factors: A comparison of the AAP/CDC case definition and the EFP/AAP classification. Int. J. Environ. Res. Public Health.

[5] Jiao J., Jing W., Si Y., Feng X., Tai B., Hu D., Lin H., Wang B., Wang C., Zheng S. (2021). The prevalence and severity of periodontal disease in Mainland China: Data from the Fourth National Oral Health Survey (2015–2016). J. Clin. Periodontol..

[6] Bakari W.N., Thiam D., Mbow N.L., Samb A., Guirassy M.L., Diallo A.M., Diouf A., Seck-Diallo A., Benoist H.M. (2021). New classification of periodontal diseases (NCPD): An application in a sub-Saharan country. BDJ Open.

[7] Tonetti M.S., Greenwell H., Kornman K.S. (2018). Staging and grading of periodontitis: Framework and proposal of a new classification and case definition. J. Periodontol..

[8] Sutthiboonyapan P., Wang H.L., Charatkulangkun O. (2020). Flowcharts for easy periodontal diagnosis based on the 2018 new periodontal classification. Clin. Adv. Periodontics.

[9] Herrera D., Sanz M., Kebschull M., Jepsen S., Sculean A., Berglundh T., Papapanou P.N., Chapple I., Tonetti M., EFP Workshop Participants and Methodological Consultant (2022). Treatment of stage IV periodontitis: The EFP S3 level clinical practice guideline. J. Clin. Periodontol..

[10] Sanz M., Herrera D., Kebschull M., Chapple I., Jepsen S., Berglundh T., Sculean A., Tonetti M.S., EFP Workshop Participants and Methodological Consultants (2020). EFP workshop participants and methodological consultants. Treatment of stage I–III periodontitis—The EFP S3 level clinical practice guideline. J. Clin. Periodontol..

[11] Schwendicke F., Krois J., Engel A.S., Seidel M., Graetz C. (2020). Long-term periodontitis treatment costs according to the 2018 classification of periodontal diseases. J. Dent..

[12] Agnese C.C.D., Schöffer C., Kantorski K.Z., Zanatta F.B., Susin C., Antoniazzi R.P. (2025). Periodontitis and oral health-related quality of life: A systematic review and meta-analysis. J. Clin. Periodontol..

[13] Raittio E., Grytten J., Lopez R., Blich C.C., Vettore M.V., Baelum V. (2025). Applying the current European periodontitis clinical practice guidelines is not feasible, even for the richest countries in the world. Community Dent. Oral Epidemiol..

[14] Mombelli A., Cionca N., Almaghlouth A. (2011). Does adjunctive antimicrobial therapy reduce the perceived need for periodontal surgery?. Periodontol. 2000.

[15] van Winkelhoff A.J., Rodenburg J.P., Goené R.J., Abbas F., Winkel E.G., de Graaff J. (1989). Metronidazole plus amoxycillin in the treatment of Actinobacillus actinomycetemcomitans-associated periodontitis. J. Clin. Periodontol..

[16] Winkel E.G., van Winkelhoff A.J., Timmerman M.F., van der Velden U., van der Weijden G.A. (2001). Amoxicillin plus metronidazole in the treatment of adult patients with periodontitis. A double-blind placebo-controlled study. J. Clin. Periodontol..

[17] Chatzopoulos G.S., Wolff L.F. (2026). Effect of adjunctive systemic antibiotics on the outcomes of non-surgical periodontal therapy: A retrospective cohort study. J. Clin. Periodontol..

[18] Botelho J., Lyra P., Nascimento G.G., Leite F.R.M., Mendes J.J., Machado V. (2025). Antibiotics in periodontal treatment: An umbrella review. Front. Cell. Infect. Microbiol..

[19] Kondreddy K., Mohanty A., Desai V., Surekha G.L., Khader A.A., Mathur S., Shetty S. (2025). Effect of adjunctive antimicrobial therapy on the clinical outcomes of non-surgical periodontal treatment: A systematic review. J. Pharm. Bioallied Sci..

[20] Trindade D., Carvalho R., Machado V., Chambrone L., Mendes J.J., Botelho J. (2023). Prevalence of periodontitis in dentate individuals between 2011 and 2020: A systematic review and meta-analysis of epidemiological studies. J. Clin. Periodontol..

[21] World Health Organization (WHO) (2024). Promoting Antimicrobial Stewardship to Tackle Antimicrobial Resistance.

[22] Shea B.J., Reeves B.C., Wells G., Thuku M., Hamel C., Moran J., Moher D., Tugwell P., Welch V., Kristjansson E. (2017). AMSTAR 2: A critical appraisal tool for systematic reviews that include randomised or non-randomised studies of healthcare interventions, or both. BMJ.

[23] Page M.J., McKenzie J.E., Bossuyt P.M., Boutron I., Hoffmann T.C., Mulrow C.D., Shamseer L., Tetzlaff J.M., Akl E.A., Brennan S.E. (2021). The PRISMA 2020 statement: An updated guideline for reporting systematic reviews. BMJ.

[24] Lin S.Y., Lin H.Y., Sun J.S., Chang J.Z. (2025). Efficacy of adjunctive periodontal interventions in non-surgical periodontal therapy for Stage III/IV Grade C periodontitis: A systematic review and network meta-analysis. Jpn. Dent. Sci. Rev..

[25] Jones O.P., Hoyle P.J. (2022). Azithromycin as an adjunct to subgingival professional mechanical plaque removal in the treatment of grade C periodontitis: A systematic review and meta-analysis. J. Periodontal Implant Sci..

[26] Pieper D., Antoine S.L., Mathes T., Neugebauer E.A., Eikermann M. (2014). Systematic review finds that overlapping reviews were not mentioned in every overview. J. Clin. Epidemiol..

[27] Guyatt G.H., Oxman A.D., Kunz R., Vist G.E., Falck-Ytter Y., Schünemann H.J. (2008). What is “quality of evidence” and why is it important for clinicians?. BMJ.

[28] Sgolastra F., Petrucci A., Ciarrocchi I., Masci C., Spadaro A. (2021). Adjunctive systemic antimicrobials in the treatment of chronic periodontitis: A systematic review and network meta-analysis. J. Periodontal Res..

[29] Teughels W., Feres M., Oud V., Martín C., Matesanz P., Herrera D. (2020). Adjunctive effect of systemic antimicrobials in periodontitis therapy: A systematic review and meta-analysis. J. Clin. Periodontol..

[30] Zandbergen D., Slot D.E., Niederman R., van der Weijden F.A. (2016). Concomitant administration of systemic amoxicillin and metronidazole versus scaling and root planing alone for treating periodontitis: A systematic review. BMC Oral Health.

[31] Stenchlakova B., Bacinsky M., Augustin M., Grendar M., Siebert T. (2026). Adjunctive systemic antimicrobials in the treatment of generalised Stage III, Grade C periodontitis: A triple-blind randomised placebo-controlled trial. Bratisl. Med. J..

[32] Herrera D., Alonso B., León R., Roldán S., Sanz M. (2008). Antimicrobial therapy in periodontitis: The use of systemic antimicrobials against the subgingival biofilm. J. Clin. Periodontol..

[33] Ramachandra S.S., Woodford V., Han P., Lee R.S.B., Ivanovski S. (2025). Systemic azithromycin as an adjunct to non-surgical subgingival instrumentation in the treatment of Stage III/IV, Grade C periodontitis: 12-month clinical, microbiological and cytokine results of a randomised controlled trial. J. Clin. Periodontol..

